# Prevention of Cerebral Embolism Progression by Emergency Surgery of the Left Atrial Myxoma

**DOI:** 10.1155/2015/151802

**Published:** 2015-04-14

**Authors:** Syuichi Tetsuka, Kunihiko Ikeguchi

**Affiliations:** ^1^Department of Neurology, Hospital of International University of Health and Welfare, 537-3 Iguchi, Nasushiobara, Tochigi 329-2763, Japan; ^2^Jichi Medical University Station Brain Clinic, 3-2-2 Idaimae, Shimotsuke, Tochigi 329-0403, Japan

## Abstract

A 21-year-old woman developed left hemiparesis during work and was hospitalized. Her National Institutes of Health Stroke Scale score was 4. Hyperintense areas in the left basal ganglia, corona radiata, and cortex of the temporal lobe were found by brain diffusion-weighted magnetic resonance imaging, indicating acute cerebral infarction. Echocardiography showed a giant mass of diameter 7 × 4 cm in the left atrium. Therefore, she was diagnosed with cerebral embolism due to a left atrial myxoma. Currently, thrombolytic therapy may continue to be effective because the embolic source may be composed of tumor tissue itself. In case of atrial myxoma, we considered that the use of tPA as emergency treatment in all patients with infarction by atrial myxoma may be questioned. Thus, cardiac tumor extraction was performed the next day after hospitalization without thrombolytic therapy. The excised myxoma measured 7 × 6 × 4 cm. The patient recovered and her neurological symptoms also improved. Furthermore, her National Institutes of Health Stroke Scale score improved to 0. Thirteen days after admission, the patient was discharged from our hospital. Cardiac myxoma is often associated with a high risk of embolic episodes, which emphasizes the need for prompt surgical excision as soon as the diagnosis is confirmed.

## 1. Introduction

Cardiac myxoma has an incidence of 0.002% among the general population and is the most common heart tumor that represents 50% of all cardiac tumors. About 75% of cardiac myxomas are located in the left atrium [1]. At least 25% of patients with left atrial myxoma present with ischemic neurological events secondary to embolism [2, 3]. The embolic source may be composed of tumor tissue itself, blood clots, or both [4, 5].

Currently, studies reporting that thrombolytic therapy with recombinant tissue plasminogen activator (tPA) in patients with sudden cerebral infarction associated with myxoma is highly effective are increasing [6–11]. However, because the embolic source may be composed of tumor tissue itself in case of atrial myxoma, we considered that the use of tPA as emergency treatment in all patients with infarction due to atrial myxoma may be questioned. In addition, the guidelines [12–14] do not discuss stroke with myxoma, and there are a few reports that emphasize the need for the prompt surgical excision of myxoma without thrombolytic therapy as soon as the diagnosis is confirmed [15, 16]. Here we present a case of a 21-year-old female with left atrial myxoma who developed an acute cerebral arterial infarction. She underwent immediate removal of the myxoma after stroke with no major enlargement of the infarct or hemorrhagic transformation without thrombolytic therapy.

## 2. Case Report

**Figure 1:**
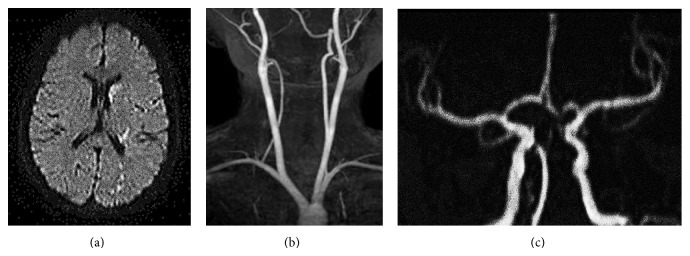
Brain MR images before cardiac tumor extraction. (a) Multiple hyperintense areas in the left basal ganglia, corona radiata, and cortex of the temporal lobe were found by diffusion-weighted MRI. (b) MR angiography did not reveal angiostenosis.

**Figure 2:**
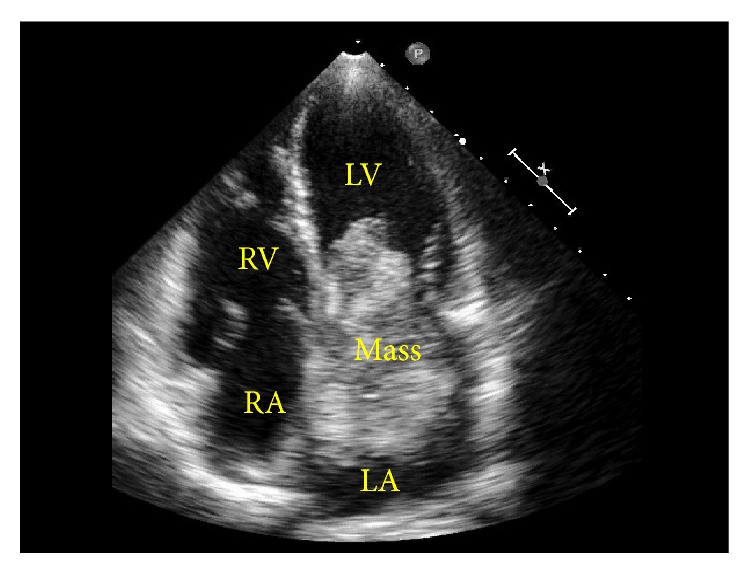
Echocardiography before surgical resection. Transesophageal four-chamber view showed a pedunculated mobile mass in the left atrium arising from the atrial septum. There was no thrombus in the left atrial appendage. LA: left atrium, LV: left ventricle, RA: right atrium, and RV: right ventricle.

A 21-year-old female presented with a migraine. For about 3 years, she at times experienced being likely to faint upon standing and dimmed vision. However, she did not show neurological dysfunction; therefore, cerebral infarction was not suspected. When she was working in standing position for a long time in the middle of the night, she started experiencing dimmed vision, labile vertigo, and hindrance in the ability to stand at her workplace. Such episodes were associated with a following numbness and weakness in the lower extremities on the left half of her body. The estimated time from the onset of symptoms to hospital presentation was about 6 h. This consequently caused the delay between neurologic symptoms onset and diagnostic of stroke in the patient. She was hospitalized in Jichi Medical University Hospital (Shimotuke, Japan) on the morning of the same day. Upon examination, her level of consciousness was alert, and she had a body weight of 47 kg, height of 160 cm, body mass index of 18, blood pressure of 84/64 mmHg, and pulse rate of 84 beats/min. A “gallop” sound was heard along with her heart sound, but her pulmonary sound was normal, and there was no pedal edema. Neurological examination revealed muscle weakness of the left upper and lower limbs (manual muscle testing; MMT 4: holds test position against slight to moderate pressure), a decrease of superficial sensation and dysesthesia of the left half of the body, and ataxia of the left upper limb. The National Institutes of Health Stroke Scale (NIHSS) score was 4. Increasing scores indicate a more severe stroke and have been shown to correlate with the size of the infarction on both CT and MRI evaluation. Many guidelines and protocols warn that administering tPA in patients with a high NIHSS score (>22) is associated with increased risk of hemorrhagic conversion. Laboratory findings showed no abnormalities except for a slight increase in C-reactive protein (1.61 mg/dL). Her electrocardiogram showed a normal sinus rhythm. Multiple hyperintense areas in the left basal ganglia, corona radiata, and cortex of the temporal lobe were found by brain diffusion-weighted magnetic resonance imaging (MRI), indicating acute cerebral infarction (Figure 1(a)). Therefore, we diagnosed the patient with cerebral embolisms. MR angiography did not reveal cervical angiostenosis (Figure 1(b)) or intracranial aneurysm (Figure 1(c)). Transthoracic 2D-echocardiography revealed a giant left atrial tumor of the diameter 7 × 4 cm attached to the atrial septum causing mitral valve prolapse that moved during diastole and occluded the mitral orifice (Figure 2).

**Figure 3:**
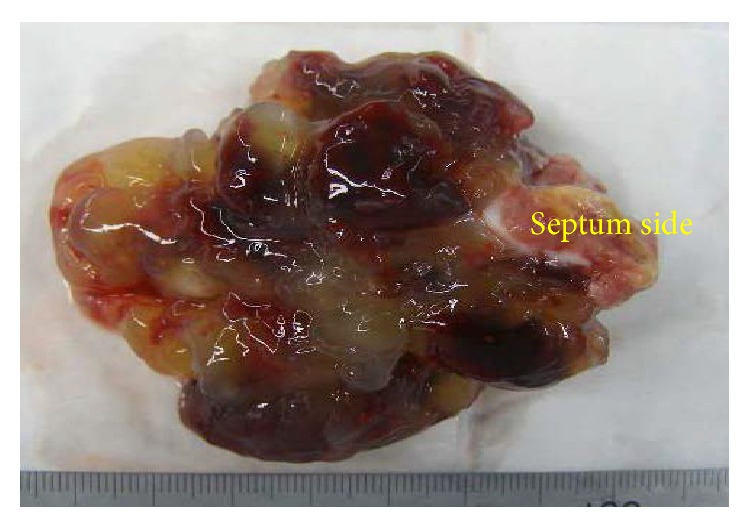
Gross pathology. The excised myxoma measured 7 × 6 × 4 cm. There was a pedicle on the septal side and the tip was papilliform.

Therefore, we diagnosed the patient with cerebral embolisms due to left atrial myxoma. The reason to not select tPA as the emergency treatment was that the estimated time from the onset of symptoms to hospital presentation was too long for patients with cerebral embolism to treat by tPA. To prevent recurrence, she underwent the resection of the myxoma with cardiopulmonary bypass and the reconstruction of the atrial septum using a bovine pericardial patch 1 day after her stroke. Gross examination showed a 7 × 6 × 4 cm brown, gray, and whitish myxoid mass (Figure 3). Pathology confirmed atrial myxoma. The patient uneventfully recovered from cardiac surgery, and her postoperative general and neurological symptoms improved. Thirteen days after admission, her NIHSS score improved to 0 and she was discharged from our hospital.

## 3. Discussion

Myxoma is a benign tumor of the heart most commonly occurring in the left atrium and is attached by a pedicle to the fossa ovalis in the atrial septum. Clinical symptoms include the classic triad of embolic, obstructive, and constitutional signs such as cerebral infarction, syncope, dyspnea, fever, weight loss, and arthralgia. In this study, the patient showed a past history of obstruction; cerebral infarction was a secondary presentation and C-reactive protein level was mildly elevated. Myxoma can generally be easily diagnosed by transthoracic echocardiography. In cases like the present where there is no risk factor for cerebrovascular disorders or atrial fibrillation, it is important to consider the possibility of myxoma as a cause of cerebral embolism despite its rarity. If time constraints and available facilities permit, transthoracic echocardiography should be performed. In spite of the benign histological character of these tumors, they can lead to unfavorable consequences. These tumors could aggravate preexisting complications or lead to sudden death due to their fragmentability or valve obstruction [17, 18].

Ischemic cerebral infarction is one of the most common as well as serious presentations of cardiac myxoma [2, 3]. However, there are no clear guidelines for immediate medical management following stroke due to atrial myxoma. The treatment of acute ischemic strokes caused by embolic atrial myxoma is controversial, largely because the embolus could be composed of the tumor itself or thrombus, adhered thrombotic material, or a combination of both [5]. It is reasonable to argue that the best possible course of action is thrombolytic therapy, depending on the composition of the emboli. Studies have reported that patients with sudden cerebral infarction associated with myxoma in whom thrombolytic therapy was highly effective and pathologic findings suggest thrombotic embolism are increasing [6–11]. But it is difficult to draw a firm conclusion from the paucity of existing data, which consists of single-case reports employing a variety of methods and the administration of different thrombolytic drugs.

On the other hand, the patient had a good outcome by emergency surgery of the left atrial myxoma without thrombolytic therapy. Such reports are rare because systemic anticoagulation required for cardiopulmonary bypass may become an issue in patients who have suffered a recent stroke, and there is a risk of hemorrhagic transformation associated with cardiopulmonary bypass in patients with a recent stroke. A large embolic stroke may become hemorrhagic and result in brain damage, particularly during the first week after stroke [19]. There are no clear guidelines in the literature indicating a safe interval from the onset of a stroke to the time of surgery; therefore, we believe the timing of surgery is still controversial and needs to be clarified. Because of the immediate diagnosis of stroke, the presence of myxomatous intracranial aneurysm and the increased risk of recurrent ischemic stroke with delayed surgery. However, the patient was taken for emergency cardiac surgery within 24 h of the stroke without such obstruction. This case suggests that, in cases without a large embolic stroke or high NIHSS score, the emergency surgery of the left atrial myxoma often gives patients with cardiogenic embolism a good outcome. Because patients with atrial myxomas are at risk of sudden death due to tumor embolism or occlusion of valve orifices, prompt surgical excision after diagnosis is mandatory. Surgery is generally curative and is associated with a perioperative mortality rate of less than 5%. Cardiac surgery with cerebral infarction has the abovementioned risk; however, there is no effective alternative to prevent cerebral embolism progression [20]. However, further epidemiological analyses in larger case studies are required before confirming its clinical utility as a treatment.

## 4. Conclusion

Thrombolytic therapy may be an exciting alternative to surgery. Cardiac myxoma has a high tendency to produce disabling neurological complications due to the risk of embolic episodes, which emphasize the need for its prompt surgical excision as soon as the diagnosis is confirmed. Moreover, in cases with a small embolism stroke and low NIHSS score, at present, we consider the emergency surgery of the left atrial myxoma to be an effective therapy for the prevention of cerebral embolism progression.
